# Is disability pension a risk indicator for future need of psychiatric healthcare or suicidal behavior among MS patients- a nationwide register study in Sweden?

**DOI:** 10.1186/s12888-015-0668-6

**Published:** 2015-11-16

**Authors:** Charlotte Björkenstam, Petter Tinghög, Philip Brenner, Ellenor Mittendorfer-Rutz, Jan Hillert, Jussi Jokinen, Kristina Alexanderson

**Affiliations:** Division of Insurance medicine, Department of Clinical Neuroscience, Karolinska Institutet, SE-171 77 Stockholm, Sweden; Division of Neuroscience, Department of Clinical Neuroscience, Karolinska Institutet, Stockholm, Sweden

**Keywords:** Multiple sclerosis, Disability pension, Suicidal behavior, Psychiatric healthcare, Sick leave

## Abstract

**Background:**

Mental disorders and suicidal behavior are common in patients with multiple sclerosis (MS), they also carry a higher risk of disability pension (DP). Our aim was to investigate if DP and other factors are associated with psychiatric disorders and suicidal behavior among MS patients, and whether DP is a stronger risk indicator among certain groups.

**Method:**

A prospective population-based cohort study with six-year follow-up (2005–2010), including 11 346 MS patients who in 2004 were aged 16–64 and lived in Sweden. Incidence rate ratios (IRR) with 95 % confidence intervals (CI) were calculated.

**Results:**

MS patients on DP had a modestly higher risk of requiring psychiatric healthcare, IRR: 1.36 (95 % CI: 1.18-1.58). MS patients with previous psychiatric healthcare had a higher IRR for both psychiatric healthcare and suicidal behavior; 2.32 (2.18-2.47) and 1.91 (1.59-2.30), respectively. DP moderated the association between sex and psychiatric healthcare, where women on DP displayed higher risk than men, X^2^ 4.74 (*p* = 0.03).

**Conclusion:**

The findings suggest that losing one’s role in work life aggravates rather than alleviates the burden of MS, as MS patients on DP seem to have a higher need for psychiatric healthcare, especially among women; which calls for extra awareness among clinicians.

## Background

Multiple sclerosis (MS) is a disabling demyelinating autoimmune disorder and is the most common cause of neurological disability among young adults [1]. Worldwide, approximately 2.5 million people are affected [2]. The etiology of MS is still largely unknown but both genetic and environmental factors have been shown to be of importance [3]. A recent study showed that as many as 62 % of MS patients of working ages are on disability pension (DP) [4]. It is also well established that the risk of developing mental disorders is higher in patients with MS [5], as is suicide attempts and suicide [6, 7]. Nevertheless, it is still unknown if MS patients on DP have a higher risk of mental disorders and suicidal behavior.

The higher risk of mental disorders in MS patients could reflect direct or indirect effects of an autoimmune disorder on the central nervous system, it could be due to medications used in the treatment, it could be due to the psychological impact of suffering from a chronic disease, or other mechanisms could be at hand [8]. Mental disorders remain under-diagnosed and undertreated in MS patients and MS patients at lower socioeconomic levels have the highest degree of comorbidity with depression [9]. The reported prevalence of depression among MS patients varies, however, widely [10]. Depression is probably the most common underlying factor for both psychiatric healthcare and suicidal behavior in these patients. Nevertheless, more knowledge is warranted on how these associations are distributed among MS patients as a basis for intervention.

There is only limited knowledge on how socio-demographics or DP status among MS patients are associated with psychiatric healthcare or with suicidal behavior. Moreover, no studies have so far investigated whether socio-demographic factors’ associations with psychiatric health are moderated by DP status among MS patients. In this study the aims were to:

- Investigate if DP and socio-demographic and clinical factors are associated with future psychiatric healthcare and suicidal behavior among MS patients.
- Analyze whether DP moderates the associations between different socio-demographics and clinical factors and psychiatric healthcare and suicidal behavior.

## Methods

### Study population

This is a population-based prospective cohort study with a six-year follow-up period; 2005–2010. We included all individuals who 31 December 2004 were 16–64 years of age, lived in Sweden, and, according to the Swedish patient register (PAR), had been diagnosed with MS (ICD-8: 340, ICD-9: 340, ICD-10: G35) sometime between 1969 and 2004. This generated 11 346 MS patients, whereof 7 990 women (70 %) and 3 356 men. PAR is held by the National Board of Health and Welfare and contains information on in-patient care since 1964 and specialized out-patient care since 2001. Our MS patients were thus selected from the in-patient care register between 1969 and 2004 and from the out-patient care register between 2001 and 2004. The unique personal identity number assigned to each Swedish citizen or permanent resident was used to link information from different registers [11].

### Exposures

*Disability pension,* which in Sweden can be granted to individuals who, due to disease or injury, have permanently reduced work capacity, even if they do not have previous income from work. DP covers approximately 65 % of the lost income, up to a limit. Information on DP in 2004 was obtained from the National Social Insurance Agency’s MiDAS database.

### Socio-demographics in 2004 and clinical factors

*Educational level* was obtained from Statistics Sweden’s Longitudinal Integration Database for Health Insurance and Labor Market Studies (LISA), and classified into: 1) ≤9 years, compulsory school, 2) 10–12 years, senior high school), and 3) >12 years, college or university.

*Age* was categorized into six groups: 16–29, 30–39, 40–49, 50–59, and 60–64.

*Country of birth,* categorized into three groups (Sweden, other Nordic countries, and the rest of the world) was also obtained from LISA, as was *marital status,* dichotomized as married/not married.

*The decade when the patient was diagnosed with MS,* was categorized into four groups: 70s (including 1969), 80s, 90s, and the 21^st^ century. Also *previous in- or outpatient specialized healthcare for psychiatric disorders* (coded according to the *International Classification of Disease* (ICD) as ICD-9: 290–319 or ICD-10: F00-F99), as main or secondary diagnosis, categorized as at least one such hospitalization or out-patient visit between 1987 and 2004 was analyzed.

### Outcomes

We analyzed two separate outcomes: psychiatric healthcare and suicidal behavior. Psychiatric healthcare was defined as having a psychiatric diagnosis in either in- or specialized out-patient care during the follow-up period 2005–2010. Suicidal behavior was also measured during 2005–2010 and included both suicide attempts and completed suicides. Information on suicide attempts was obtained from PAR and defined as having a diagnosis of self-inflicted harm (ICD-10: X60-X84). Suicide was defined as having suicide as the underlying cause of death in the Causes of Death Register (ICD-10: X60-X84) or as death with undetermined intent (ICD-10: Y10-Y34). With our definition of suicide including deaths with undetermined intent, our aim was to reduce spatial and secular trends in detecting and classifying suicide [12].

### Statistical analyses

We used Poisson regression analyses to estimate associations between DP and different socio-demographics and clinical factors and the two outcomes; psychiatric healthcare and suicidal behavior. As a measure of the relative occurrence of psychiatric healthcare and suicidal behavior we used the incidence rate ratios (IRR) and adjusted for all socio-demographics in 2004 and the clinical factors (mentioned above). By adding up the years the individuals were alive and living in Sweden (from the LISA database) during the follow-up period we assessed person-years at risk. All individuals were followed from 1 January 2005 until the end of follow-up or until they experienced the respective event, emigrated, or died, whichever came first. SAS GENMOD procedure was used to calculate IRR and 95 % confidence intervals (CIs) for psychiatric healthcare and suicidal behavior, respectively.

We also performed a sensitivity analysis where we excluded MS patients who had been on DP prior to 2003. In this analysis we compared MS patients who only recently (in 2003 or 2004) had been granted DP to MS patients not on DP, and censoring MS patients who were granted DP during followup. Further, we ran an extra analysis where we excluded MS patients with a history of psychiatric care.

We constructed one model including all main effects and six interaction terms of interest to determine whether different background factors moderated the association between DP and the outcomes. Effect modifications were evaluated with Wald X^2^ tests. Statistical significance was defined as p < 0.05. All analyses were carried out in the statistical software SAS 9.1 (SAS Institute Inc. Cary, NC, USA).

## Ethics statement

The study population was based on linkage of several public national registers. Ethical vetting is always required when using register data in Sweden. The ethical vetting is performed by regional ethical review boards and the risk appraisal associated with the Law on Public Disclosure and Secrecy is done by data owners. The ethical review boards can however waive the requirement to consult the data subjects (or in case of minors/children the next of kin, careers or guardians) directly to obtain their informed consent, and will often do so if the research is supported by the ethical review board and the data has already been collected in some other context. According to these standards in Sweden this project has been evaluated and approved by the Regional Ethical Review Board of Karolinska Institutet, Stockholm, Sweden.

## Results

In our cohort of 11 346 MS patients of working ages (70 % women), 6 399 were on DP at baseline 2004 (Table 1). As expected, it was more common among MS patients on DP to be older, 21 % was in the oldest age group (60–64 years), compared to 6 % among MS patients not on DP. Low educational level was also more common among MS patients on DP in 2004, 25 % compared to 12 % among MS patients who were not on DP. In total, 1 104 individuals (10 %) received psychiatric healthcare and 118 individuals (1 %) had suicidal behavior during the follow-up period, 2005–2010. The rate of women was very similar in the two outcome groups as in the whole cohort, 72 % and 71 % related to future psychiatric health care and suicidal behavior, respectively, compared to 70 % women in total. Among MS patients on DP in 2004, 11 % had psychiatric healthcare during follow-up compared to 9 % of those not on DP. It was more common to have had psychiatric healthcare before 2005 in the two outcome groups, 53 % of the psychiatric healthcare group and 50 % of the suicidal behavior group compared to 14 % among MS patients not on DP. Previous psychiatric health care was also more common among MS patients on DP, 25 %, compared to 14 % among patients not on DP.

**Table 1:** Descriptive data (n and %) of the cohort of 11 346 patients with multiple sclerosis (MS), among all, those not on disability pension (DP) and on DP in 2004, regarding future psychiatric healthcare and suicidal behavior, respectively

				Psychiatric healthcare in 2005-2010			Suicidal behavior 2005-2010		
	DP	No DP	Total	DP	No DP	Total	DP	No DP	Total
All	6399 (56)	4947 (44)	11 346	678 (61)	426 (39)	1 104	78 (66)	40 (34)	118
Sex									
Women	4659 (58)	3331 (42)	7 990	522 (65)	275 (35)	797	57 (68)	27 (32)	84
Men	1740 (52)	1616 (48)	3 356	156 (51)	151 (49)	307	21 (62)	13 (38)	34
Decade of MS Diagnosis									
70s	404 (68)	191 (32)	595	35 (70)	15 (30)	50	8 (100)	0 (0)	8
80s	1192 (77)	351 (23)	1 543	115 (78)	33 (22)	148	10 (71)	4 (29)	14
90s	1986 (74)	708 (26)	2 694	213 (81)	49 (19)	262	23 (82)	5 (18)	28
21st century	2817 (43)	3697 (57)	6 514	315 (49)	329 (51)	644	37 (54)	31 (46)	68
Age 2004									
16-29	121 (16)	644 (84)	765	37 (33)	76 (67)	113	3 (37)	8 (73)	11
30-39	657 (31)	1464 (69)	2 121	94 (42)	129 (58)	223	8 (44)	10 (56)	18
40-49	1536 (51)	1474 (49)	3 010	201 (62)	123 (38)	324	25 (61)	16 (39)	41
50-59	2746 (72)	1087 (28)	3 833	254 (76)	79 (24)	333	30 (86)	5 (14)	35
60-64	1339 (83)	278 (17)	1 617	92 (83)	19 (17)	111	12 (92)	1 (8)	13
Psychiatric diagnosis before 2005									
Yes	1594 (70)	699 (30)	2 293	403 (69)	177 (31)	580	46 (78)	13 (22)	59
No	4805 (53)	4248 (47)	9 053	275 (52)	249 (48**)**	524	32 (54)	27 (46)	59
Educational level 2004									
≤9 years	1580 (73)	599 (27)	2 179	150 (70)	63 (30)	213	16 (84)	3 (16)	19
10-12 years	3504 (57)	2654 (43)	6 158	383 (62)	233 (38)	616	56 (64)	31 (36)	87
>12 years	1315 (44)	1694 (56)	3 009	145 (53)	130 (47)	275	6 (50)	6 (50)	12
Country of birth									
Sweden	5831 (56)	4496 (44)	10 327	599 (62)	373 (38)	972	71 (67)	35 (33)	106
Other Nordic	222 (64)	126 (36)	348	28 (72)	11 (28)	39	4 (67)	2 (33)	6
Other World	346 (52)	325 (48)	671	51 (55)	42 (45)	93	3 (50)	3 (50)	6
Marital Status 2004									
Married	3123 (57)	2371 (43)	5 494	239 (58)	176 (42)	415	24 (65)	13 (35)	37
Unmarried	3276 (56)	2576 (44)	5 852	439 (64)	250 (36)	689	54 (67)	27 (33)	81

When we calculated multi-adjusted IRR for DP and the different socio-demographics and clinical factors, previous psychiatric healthcare was statistically significant for both outcomes; psychiatric healthcare IRR: 2.32 (95 % CI: 2.18-2.47) and suicidal behavior IRR: 1.91 (1.59-2.30) (Table 2). MS patients on DP displayed higher IRR for psychiatric healthcare: 1.36 (1.18-1.58). We found a tendency towards a higher risk for suicidal behavior, however, not statistically significant IRR: 1.46 (0.95-2.26). Individuals born outside of the Nordic countries had higher risk for psychiatric healthcare; IRR: 1.29 (1.08-1.54) as did the youngest patients, aged 16–29; IRR: 1.77 (1.49-2.10). Unmarried individuals displayed higher risk for both psychiatric healthcare IRR: 1.14 (1.07-1.22) as well as for suicidal behavior IRR: 1.32 (1.08-1.62).

**Table 2:** Adjusted^a^ incidence rate ratios (IRR) with 95 % confidence intervals (CI) for psychiatric healthcare and suicidal behavior, respectively, during five-year follow up among 11 346 MS patients of working ages

		Psychiatric healthcare	Suicidal behavior
		IRR (95 % CI)	IRR (95 % CI)
Sex	F	0.95 (0.89-1.01)	0.98 (0.80-1.20)
	M	1 (REF)	1 (REF)
DP 2004	Yes	1.36 (1.18-1.58)	1.46 (0.95-2.26)
	No	1 (REF)	1 (REF)
Country of birth	Nordic	0.90 (0.72-1.13)	1.36 (0.74-2.49)
	World	1.29 (1.08-1.54)	0.85 (0.46-1.55)
	Swe	1 (REF)	1 (REF)
Age 2004	16-29	1.77 (1.49-2.10)	1.44 (0.84-2.48)
	30-39	1.19 (1.05-1.35)	0.88 (0.57-1.35)
	40-49	1.07 (0.96-1.19)	1.23 (0.89-1.70)
	50-59	0.77 (0.69-0.86)	0.85 (0.60-1.21)
	60-64	1 (REF)	1 (REF)
Decade of MS	1970s	0.98 (0.78-1.22)	1.36 (0.78-2.39)
Diagnosis	1980s	1.02 (0.89-1.19)	0.75 (0.47-1.19)
	1990s	0.94 (0.83-1.07)	0.90 (0.62-1.30)
	2000s	1 (REF)	1 (REF)
Marital status 2004	Unmarried	1.14 (1.07-1.22)	1.32 (1.08-1.62)
	Married	1 (REF)	1 (REF)
Education 2004	≤9 years	0.96 (0.86-1.07)	0.95 (0.65-1.39)
	10-12 years	1.01 (0.93-1.09)	1.83 (1.38-2.44)
	>12 years	1 (REF)	1 (REF)
Previous psychiatric healthcare	Yes	2.32 (2.18-2.47)	1.91 (1.59-2.30)
	No	1 (REF)	1 (REF)

^a^Adjusted for all covariates

In our sensitivity analyses, where we only included MS patients who recently were granted DP, we found similar risk estimates, IRR: 1.41(1.15-1.73) for psychiatric health care and IRR: 1.06 (0.46-2.43) for suicidal behavior (data not shown). Further, excluding MS patients with a history of psychiatric care yielded an IRR of 1.66 (1.27-2.16) for psychiatric health care (data not shown).

We performed effect-modification analyses to study if the IRRs were higher among people on DP regarding our socio-demographics and clinical factors (Tables 3 and 4). DP moderated the association between sex and psychiatric healthcare, where DP seemed to increase the risk for psychiatric healthcare more among women than among men, df 1,X^2^ 4.74 (*p* = 0.03). No other significant effect-modifications were found (p < 0.05).

**Table 3:** Incidence rate ratios (IRR) with 95 % confidence intervals (CI) for psychiatric healthcare in 2005–2010 among 11 346 MS patients not on disability pension (DP) and on DP in 2004, respectively, regarding different background factors

IRR for psychiatric health care^a^				
		No DP	DP	
		IRR (95 % CI)	IRR (95 % CI)	Effect modifications X2 (p-values)
Sex	F	1.00 (0.90-1.11)	1.15 (1.05-1.26)	df 1, 4.74 (0.03)
	M	1 (REF)	1 (REF)	
Country of birth	Nordic	1.02 (0.67-1.55)	0.95 (0.72-1.25)	df 2, 2.19 (0.33)
	World	0.91 (0.68-1.21)	1.09 (0.87-1.37)	
	Swe	1 (REF)	1 (REF)	
Age group 2004	16-29	1.30 (1.03-1.65)	1.65 (1.26-2.17)	df 4, 4.69 (0.32)
	30-39	1.12 (0.93-1.36)	1.09 (0.90-1.31)	
	40-49	1.18 (0.97-1.42)	1.08 (0.93-1.24)	
	50-59	0.97 (0.78-1.20)	0.84 (0.73-0.96)	
	60-64	1 (REF)	1 (REF)	
Decade of MS diagnosis	1970s	1.20 (0.78-1.84)	0.87 (0.67-1.13)	df 3, 3.83 (0.28)
	1980s	0.84 (0.62-1.13)	0.89 (0.75-1.05)	
	1990s	0.94 (0.72-1.23)	0.97 (0.84-1.12)	
	2000s	1 (REF)	1 (REF)	
Marital status 2004	Not married	1 (REF)	1 (REF)	df 1, 1.05 (0.30)
	Married	0.96 (0.86-1.06)	1.02 (0.95-1.11)	
Education 2004	≤9 years	1.10 (0.94-1.28)	1.12 (0.99-1.27)	df 2, 4.98 (0.08)
	10-12 years	1.02 (0.84-1.22)	0.83 (0.73-0.94)	
	>12 years	1 (REF)	1 (REF)	

^a^The two models are adjusted for all main effects and the 6 interaction terms (i.e., effect-modifications) of interest

**Table 4:** Incidence rate ratios (IRR) with 95 % confidence intervals (CI) for future suicidal behavior (in 2005–2010) among 11 346 MS patients not on disability pension (DP) and on DP in 2004, respectively, regarding different background factors

IRR for suicidal behavior^a^				
		No DP	DP	
	Level1	IRR (95 % CI)	IRR (95 % CI)	Effect modifications X2 (p-values)
Sex	Female	1.01 (0.72-1.41)	0.97 (0.75-1.25)	df 1, 0.07 (0.80)
	Male	1 (REF)	1 (REF)	
Country of birth	Nordic	1.44 (0.52-4.00)	1.38 (0.64-2.96)	df 2, 0.48 (0.79)
	World	0.95 (0.39-2.36)	0.75 (0.33-1.72)	
	Sweden	1 (REF)	1 (REF)	
Age group 2004	16-29	1.61 (0.75-3.48)	1.43 (0.55-3.66)	df 4, 1.34 (0.86)
	30-39	0.95 (0.39-2.36)	0.88 (0.47-1.65)	
	40-49	1.48 (0.80-2.74)	1.13 (0.72-1.77)	
	50-59	0.73 (0.31-1.69)	0.91 (0.59-1.38)	
	60-64	1 (REF)	1 (REF)	
Decade of diagnosis	1970s	0.00 (0.00-0.00)	1.70 (0.95-3.03)	df 3, 5.76 (0.12)
	1980s	361.55 (89.46-1461.19)	0.60 (0.35-1.03)	
	1990s	224.66 (149.36-337.92)	0.88 (0.58-1.32)	
	2000s	1 (REF)	1 (REF)	
Marital status	Not married	1 (REF)	1 (REF)	df 1, 0.13 (0.71)
2004	Married	1.25 (0.88-1.77)	1.35 (1.05-1.73)	
Education 2004	<=9 years	0.83 (0.37-1.88)	1.00 (0.64-1.58)	df 2, 0.16 (0.92)
	10-12 years	1.92 (1.14-3.23)	1.84 (1.28-2.66)	
	>12 years	1 (REF)	1 (REF)	

^a^The two models are adjusted for all main effects and the 6 interaction terms (i.e., effect-modifications) of interest

## Discussion

In this population-based prospective cohort study of 11 346 MS patients of working ages, we found that patients who were on DP in 2004 had a higher risk for psychiatric healthcare during the six-year follow up, whereas MS patients with previous psychiatric healthcare had a higher risk for both psychiatric healthcare and suicidal behavior. DP moderated (statistically significant) only the association between sex and psychiatric healthcare, where women on DP had higher risk for psychiatric healthcare during the follow-up than men.

The strengths of this study include the population-based prospective cohort design, the large number of MS patients, and the use of high-quality and nationwide register data with high completeness and validity and no loss to follow up [13]. Our study also has some limitations. As out-patient visits were not included in the Patient register before 2001, the assumed decade of diagnosis might be later than the true one for those diagnosed before 2001, as many MS patients are not hospitalized and especially not at the time of diagnosis. We analyzed psychiatric healthcare (in-patient and specialized out-patient care), however, this does not cover all mental disorders, as primary health care visits are not included in the patient register. As this means that only the more severe mental disorders are included, this can be seen as both a limitation and as a strength. Furthermore, for the same reason, all suicidal behaviors are not noted in the registers since some acts of serious self-harming behavior do not lead to hospitalizations, specialized healthcare, or death. Moreover, some of the MS patients had been diagnosed a long time ago or been granted DP a long time ago. As the risks of suicide among MS patients are highest during the first year after diagnosis [14] and many of our patients were diagnosed several years prior to 2004, this cohort could be seen as there was a ‘health selection’ of our study population regarding the studied outcomes, and thus an underestimation of the results. In the same manner, patients diagnosed earlier had a longer exposure time for psychiatric health and suicidal behavior care prior to the start of follow-up.

Here we compared MS patients on DP in 2004 with patients not on DP in 2004. However, the latter patients could have been granted DP during the follow-up that also means a possible underestimation of risks. Even so, MS patients on DP in 2004, showed higher IRR for psychiatric healthcare, but not statistically significant higher IRRs for suicidal behavior. MS patients with a history of psychiatric ill health or suicide attempts might have a higher probability of being granted DP. This fact could in itself lead to higher risk of requiring specialized psychiatric healthcare after DP. We controlled for decade of diagnosis and previous specialized psychiatric healthcare in an attempt to reduce confounding, although these confounders are pretty obtuse which calls for cautious interpretation of our results and for further studies to compare these two groups as the residual confounding may be increased.

As we in this study lack data on cognitive and physical impairment, which are common consequences of MS [15, 16], one might argue that level of impairment could be responsible for both the probability of being granted DP and for the need of psychiatric health care. Although, the plausibility of this interpretation rests on the assumption that there is a positive association between impairment and need of psychiatric health care even after adjusting for prior psychiatric health care. This, however, is a questionable assumption since most longitudinal studies in which baseline psychiatric morbidity has been taken into account have not been able to reveal any associations between level of impairment and psychiatric morbidity [15–17].

Being on DP means that you are marginalized from the labor market, which might result in isolation, loss of meaning, change of life style, and loneliness that can affect the risk of suicidal behavior [18, 19]. It could, on the other hand, be argued that paid work among MS patients may involve stress that could increase the risk of mental disorders. In this study, the former seems to be true regarding psychiatric healthcare, and especially among women. A recent Swedish register study found a higher risk of premature death among young adults (19–23 years) on DP, but only those with mental DP diagnoses had a significantly elevated risk for suicide [18].

Another Swedish study on trajectories of suicide attempt risks before and after granting of DP in young people, found a higher risk of suicide attempt up until the granting of DP, after which the risk decreased [20]. In our cohort of MS patients of all working ages, those on DP indicated higher risk for suicidal behavior (though not statistically significant), and “only” 118 MS patients had suicidal behavior during follow up in our study. Our data on this is thus limited and consequently our estimates had rather broad CIs. Even so, when analyzing psychiatric healthcare as outcome - an outcome related to suicidal behavior – the cohort included 1 104 individuals and received similar estimates.

Not being married was one of the two factors that displayed higher risk for both outcomes. Unmarried individuals in the general population have previously been shown to have higher suicide risk, which is in line with our results [21]. A Danish study evaluated the probability of MS patients to remain married or in a relationship with the same partner after MS onset [22]. The cumulative probability of remaining in the same relationship five years after onset was 86 % in MS patients vs. 89 % in controls [22]. If the unmarried patients in our study had been previously married/cohabiting needs to be further investigated. Having prior psychiatric healthcare was the other factor that entailed higher risk for both our outcomes. As mental disorder is the main risk factor for suicide [23], our result was expected. The most severe consequence of depression is suicide [23]. Around 30 % of MS patients have reported suicide ideations [24]. A previous Swedish study found the suicide risk to be twice as high among MS patients as in the general population [14]. In this study, we did not compare suicidal behavior among MS patients with that in the general population, but compared suicidal behavior among MS patients with different socio-demographic and DP status in one year, 2004.

One general explanation of the association between lower educational level and suicidal behavior is the underlying elevated risk for mental disorders among less educated individuals [24]. In the general population it seems that mental disorder is a factor on the causal pathway between lower socio-economic position and suicidal behavior [25]. However, we found no such clear results for educational level for psychiatric healthcare or for suicidal behavior, probably as we controlled for prior psychiatric healthcare.

Existing literature on the general population has shown that women have higher risk for psychiatric healthcare [26], something we did not find in this cohort. Two previous studies found that men diagnosed with MS have a lower quality of life than women [27, 28]. Depression in MS patients has been suggested to be both part of the disease itself and to be a psychosocial reaction to the disease [29]. Estimations of depression prevalence among MS patients have fluctuated between 25-30 %, nevertheless, only 25 % of those patients received adequate antidepressant treatment from her/his neurologist [3]. The presence of depression does, however, not correlate well with the severity of the MS disease [30]. Depression impacts functional status and depressed MS patients perform more poorly on tests of cognitive function [31] which means that if depression is not optimally treated the risk of DP is higher.

DP only moderated the association between sex and psychiatric healthcare, which means that DP may be worse for women than for men regarding IRR for psychiatric healthcare. This finding needs, however, to be studied further.

## Conclusions

In conclusion, our results suggest that MS patients on DP have higher future risk of requiring psychiatric healthcare than MS patients without DP, and especially younger patients are at risk. No significant higher risk was established in regard to suicidal behavior. DP also seems to involve higher risk for psychiatric healthcare among women. These findings suggest that losing one’s role in work life aggravates rather than alleviates the burden of MS. Extra awareness among clinicians regarding MS patients on DP may be called for.

## Abbreviations

DP: Disability pension
MS: Multiple sclerosis
